# A systematic policy approach to changing the food system and physical activity environments to prevent obesity

**DOI:** 10.1186/1743-8462-5-13

**Published:** 2008-06-05

**Authors:** Gary Sacks, Boyd A Swinburn, Mark A Lawrence

**Affiliations:** 1 School of Exercise and Nutrition Sciences, Deakin University, Burwood, Australia; 2 WHO Collaborating Centre for Obesity Prevention, School of Exercise and Nutrition Sciences, Deakin University, 221 Burwood Highway, Burwood, VIC 3125, Australia

## Abstract

As obesity prevention becomes an increasing health priority in many countries, including Australia and New Zealand, the challenge that governments are now facing is how to adopt a systematic policy approach to increase healthy eating and regular physical activity. This article sets out a structure for systematically identifying areas for obesity prevention policy action across the food system and full range of physical activity environments. Areas amenable to policy intervention can be systematically identified by considering policy opportunities for each level of governance (local, state, national, international and organisational) in each sector of the food system (primary production, food processing, distribution, marketing, retail, catering and food service) and each sector that influences physical activity environments (infrastructure and planning, education, employment, transport, sport and recreation). Analysis grids are used to illustrate, in a structured fashion, the broad array of areas amenable to legal and regulatory intervention across all levels of governance and all relevant sectors. In the Australian context, potential regulatory policy intervention areas are widespread throughout the food system, e.g., land-use zoning (primary production within local government), food safety (food processing within state government), food labelling (retail within national government). Policy areas for influencing physical activity are predominantly local and state government responsibilities including, for example, walking and cycling environments (infrastructure and planning sector) and physical activity education in schools (education sector). The analysis structure presented in this article provides a tool to systematically identify policy gaps, barriers and opportunities for obesity prevention, as part of the process of developing and implementing a comprehensive obesity prevention strategy. It also serves to highlight the need for a coordinated approach to policy development and implementation across all levels of government in order to ensure complementary policy action.

## Background

The prevalence of obesity has increased to such an extent in recent decades that it has been recognised as a public health crisis in many countries, including Australia and New Zealand [1,2]. The Australian National Health and Medical Research Council (NHMRC) has identified obesity as a priority health issue for the current triennium [3] and the New Zealand government has identified the reduction of obesity as one of its population health objectives [4].

Reliance on education and treatment strategies alone is unlikely to be sufficiently effective or sustainable to stem the obesity epidemic, and there is widespread agreement that major changes to the current obesogenic environments will also be necessary [5]. Such environmental changes often need to be driven by policy, for example, increasing the opportunities for active transport (physical environment), making healthy food choices more affordable (economic environment) and changing attitudes about food marketing to children (socio-cultural environment) are unlikely to be successful without the backing of supportive policy [6]. Policy approaches are also highly appropriate for reaching multiple sectors of the community, including socio-economically disadvantaged populations where obesity levels are disproportionately high [7,8]. These reasons, along with the need for potent, sustainable and cost-effective strategies to reduce obesity prevalence all point towards the importance of policy approaches [9].

In recent years, many authors have discussed policy options available to government to prevent obesity. For example, French, Story and Jeffery [10] and Nestle and Jacobson [11] list potential environmental strategies and policy recommendations for reducing the prevalence of obesity, and Hayne, Moran and Ford [12] suggest regulatory levers worthy of consideration. While the lists generated by these authors are useful in showing the wide range of policy options available, they do not necessarily reflect a systematic approach. In order to ensure that obesity prevention policies are logical and coherent and that no major policy gaps or barriers are overlooked, we argue that it is valuable to start with a systematic scan of opportunities for intervention. A systematic approach will also ensure that synergies among policy actions can be identified and isolated policy actions that might be inconsistent with overall policy objectives can be avoided.

This article sets out a structure for systematically identifying areas for obesity prevention policy action across the food system and full range of physical activity environments, and across all levels of government. Within this structure, the article then categorises a selection of obesity prevention policy areas identified in the literature as they apply to the Australian context. In so doing, this article illustrates and classifies the broad array of legal and regulatory interventions that can be used to alter the food system and physical environments to help prevent obesity.

## Methods

A literature search was conducted to gain perspective on the previously recognised obesity prevention policy actions that focus on changes to the food system and physical activity environments. The search aimed to locate a range of articles referring to obesity prevention policies in medical, economics, policy and law journals as well as in the 'grey' literature (government and non-government organisation reports). A combination of 'Overweight', 'Obesity' and 'Social Control, Formal' were used as MeSH (Medical Subject Headings) search terms in the 'Medline' database, while a combination of 'Obesity' and 'Policy' were used as search terms in the 'EconLIT' and 'Business Source Premier' databases. The authors were referred to other scientific literature and relevant grey literature by their contacts in the fields of obesity prevention, public health law and public health nutrition.

In reviewing the policy action examples from the literature, we confined our analysis to laws and regulations, including formal written codes and decisions that bear legal authority. In so doing, we aim to highlight the wide array of legal intervention areas available, supporting the views of authors such as Nestle [13] and Gostin [14] who believe that legal and regulatory approaches have thus far been under-utilised in obesity prevention efforts. In our definition of laws and regulations, we included, for example, government policies or individual school policies on the types of food which may be sold in schools because these are codified and enforceable but we did not include non-enforceable guidelines or health promotion programs on school food. Also, we included rule-based economic interventions, such as taxes and subsidies, but not funding allocations for health promotion programs. Our scope included institutional policies within government agencies, non-governmental organisations and the private sector because these were considered rules with a degree of enforceability, and could be considered to complement the policy interventions at a government level. While we note the importance of other policy instruments (such as service delivery, programs and advocacy) available to governments and other specific interventions that are not enforceable, such as guidelines, professional practice and social norms [15], we have not focused on these specific options.

The discussions, issues and examples sourced from the relevant literature were categorised into policy areas. In this context, we define a 'policy area' as a content area in which enforceable rules could reasonably be implemented as specific policy interventions. In identifying these policy areas we recognise that specific policy interventions (e.g., legislation, regulations, other enforceable rules or policies) which could be applied in each policy area would still need to be defined further, taking into account the constraints and peculiarities of the particular policy environment. We classified policy areas under particular jurisdictions or levels of government based on the jurisdiction that typically has responsibility for administering policies in that area.

In areas where policies are enacted and administered at one level but enforced at a lower level of government, such as aspects of food safety in Australia where local governments typically enforce laws made at state level, the policy area was classified under the level of government at which the policy is administered.

## Classifying the policy areas across two dimensions

Once we had identified the set of policy areas for preventing obesity through changes to the food system and physical activity environments, we classified the policy areas across two dimensions: the level of governance that is primarily responsible for administering the policy action, and the sector to which the policy action applies most directly. Adoption of these two dimensions facilitates a practical and systematic approach to analysing the policy environment and mapping potential policy intervention areas, and is similar to the approach taken by Schmid et al [15] in outlining a conceptual framework for public policy related to physical activity and Lawrence [16] in outlining public policy opportunities in relation to nutrition.

### Multiple levels of governance

The first dimension of analysis recognises that multiple levels of governance are responsible for developing and implementing policy interventions. The levels of governance will vary from country to country so, for example, in Australia they include local government, state government, national government, as well as international governance (acknowledging that the policies of international organisations, such as the World Trade Organisation [17], can have significant bearing on the affairs of nation states). This dimension also incorporates the policies of organisations, such as government agencies, non-governmental organisations and the private sector, that may be used as tools for (or serve as barriers to) obesity prevention.

### Inter-sectoral analysis

The second dimension of analysis recognises that the health sector has only limited influence over environmental determinants and individual behaviours that contribute to obesity. Instead, the health sector's role and responsibility is confined principally to obesity care and treatment and certain promotion and education activities. It is only by analysing the policy actions of other (non-health) sectors in government and society more broadly that a comprehensive approach to obesity prevention can be developed. This approach draws on the 'healthy public policy' strategy proposed by the World Health Organization [18], and places responsibility on policy-makers in all government sectors to be accountable for the obesity impact of their policy decisions.

We considered the particular sectors to include in the analyses of the food system and physical activity environments to be quite distinct from each other. Accordingly, a separate sectoral analysis was required for each of the food system and physical activity environments and these are discussed in the sections below.

## Policy actions that influence the food system

In order to systematically analyse the policy actions that influence the food system, it is necessary to consider all sub-components of the food system, including primary production (e.g., agriculture and fishing) and the inputs to primary production (e.g., fertilisers, pesticides); food processing (e.g., dairies, abattoirs, canners, brewers); distribution (e.g., logistics, importers, exporters); marketing; retail (e.g., supermarkets, marketplaces); and catering and food service (e.g., restaurants, schools, hospitals) (adapted from [19]). As depicted in Table 1, these sectors comprise one dimension with which to analyse policies to influence the food system, with the level of governance on the other dimension. In this way, Table 1 can be used to consider the influence that the policy actions of each level of governance have on each component of the food system (e.g., the influence of national government policy with respect to primary production). The intention of obesity prevention policy change in these areas is typically to alter the food environment such that healthier choices are the easier choices for individuals.

**Table 1:** 'Policy areas' that influence the food system (related to Australian context)

**Sector**	**Level of governance**				
	**Local Government**	**State Government**	**National Government**	**International**	**Organisational**
**Primary production**	• Land-use management	• Primary production subsidies and taxes	• Primary production subsidies and taxes	• Primary production subsidies and taxes	
	• Community gardens				

**Food processing**		• Food safety	• Product composition standards		• Product composition standards

**Distribution**		• Food transport	• Importation restrictions, subsidies and taxes	• Trade arrangements	
			• Quarantine		

**Marketing**		• Marketing to children	• Marketing to children	• Marketing to children	• Marketing to children
		• Marketing practices in schools	• Nutrient content disclosures in marketing material		
			• Consumer protection (e.g., misleading advertising)		

**Retail**	• Land-use management	• Products sold in schools	• Nutrition labelling	• Nutrition labelling	• Product placement in stores
	• Density of local fresh food retailers		• Health claims on food products	• Health claims on food products	
	• Density of fast food outlets		• Incentive system for welfare recipients to buy healthy food		
			• Food taxes/subsidies		

**Catering/Food service**		• Nutrition information in restaurants			• School food policies
		• Food safety			• Standards for food served in workplaces
					• Food procurement policies

The examples shown in Table 1 represent a selection of obesity prevention policy areas related to the food system in Australia, as identified in the literature [10-12,17,20-25], for which laws and regulations are potential policy instruments. In this context, the policy areas represent potential regulatory intervention points for shaping the food system to prevent obesity. It should be noted that the items in Table 1 are intended to be illustrative only and do not represent a complete set of potential policy areas in this domain. While the policy areas we have identified are drawn from the literature, we did not make a judgement or take into account the level of evidence supporting the likely effectiveness of interventions in these areas. This sort of evaluation of the policy options identified here, using methodologies such as that used in a previous study evaluating the cost-effectiveness of various interventions [26], would be a logical next step in the process of developing a comprehensive obesity strategy.

In populating the table it was noted that some issues, such as restricting marketing of foods to children, can be influenced by the policies of multiple levels of government as well as the policies of corporate organisations and industry bodies. In these cases, the policy area was placed within multiple cells in the table to reflect the multiple areas for potential policy action.

In reviewing the populated tables for a particular country or community, it is worth considering the implications of parts of the analysis grid that are 'empty'. Typically, boxes are 'empty' because a particular level of government does not have jurisdiction to influence a particular sector, e.g., local government typically has no influence on broadcast food marketing, with the 'empty' box indicating there are no potential policy interventions in that sector for that level of government. However, the advantage of using these analysis grids as a systematic scanning tool to identify policy options is that presence of 'empty' boxes may prompt policy analysts to identify previously unrecognised policy opportunities. For example, perhaps local governments have a role to play in restricting marketing of unhealthy food through restrictions on the placement of billboards in the community.

## Policy actions that influence physical activity environments

Obesity prevention policies targeting physical activity environments will seek to alter the environment to make increased levels of physical activity and decreased levels of sedentariness the easy choices for the population. In order to have an influence, policies will need to target the sectors that control the environments within which physical activity predominantly occurs. Physical activity settings are well-documented [27,28] and can be readily translated into sectors to which policy can be targeted, including infrastructure and planning, education, employment, transport and sport and recreation (refer to Table 2). By examining these sectors on one dimension, with the level of governance on the other dimension, Table 2 can be used to consider how the policies of each level of governance can be used to influence the environment to increase the level of physical activity and decrease sedentary behaviour of the population.

**Table 2:** 'Policy areas' that influence physical activity environments (related to Australian context)

**Sector**	**Level of governance**				
	**Local Government**	**State Government**	**National Government**	**International**	**Organisational**
**Infrastructure and planning**	• Land use management (zoning)	• Urban planning	• Roads		
	• Walking environment	• Roads			
	• Cycling environment				

**Education**		• Physical education in schools			• School policies on physical education, physical activity and sport
		• Facilities for physical activity in schools			

**Employment**		• Building design standards			

**Transport**	• Public transport	• Public transport	• Taxation policies on cars	• Trade arrangements on motor vehicles	• School travel policies
	• Parking restrictions	• Traffic control			
	• Traffic control		• Taxation incentives for using public transport		

**Sport and recreation**	• Facilities for physical activity -built structures	• Public liability			
		• Access of general community to school sport facilities			
	• Facilities for physical activity -open spaces				
	• Public liability				

The examples shown in Table 2 represent a selection of obesity prevention policy areas, as identified in the literature [10-12,20,21,23,24], that can be used to influence physical activity environments in the Australian context. The policy areas in the table represent potential regulatory intervention points for shaping the physical activity environment to prevent obesity. Once again, it should be noted that the items in Table 2 are intended to be illustrative only and do not represent a complete set of potential obesity prevention policy areas in this domain, nor are they meant to indicate the potential effectiveness of interventions in the area.

It is notable in Table 2 that, in the Australian context, the majority of policy areas influencing physical activity environments fall under the responsibility of local and state governments. There appears to be less of a role for the national government and international policy actions in this area, which contrasts with the analysis of the food environment (Table 1) that shows a number of policy areas which would be amenable to national and international policy actions. It is also noted that we did not identify policy areas related to sedentary behaviour (e.g., television viewing and online gaming) that would be amenable to the application of enforceable rules.

## Discussion

The analysis grids presented in this article provide a tool for systematically scanning for policy opportunities to change the food system and physical activity environments to prevent obesity. The use of this tool as part of developing a comprehensive obesity strategy may help reduce the risks of the ad hoc approaches often adopted in addressing this issue by ensuring that all major policy gaps and opportunities are identified for subsequent evaluation. This type of analysis can be applied at the level of individual local, state and/or national governments, and can be used by a wide array of groups including policy developers and analysts, advocacy groups and researchers.

In examining illustrative examples of potential legal and regulatory intervention areas in the Australian context, the policy areas identified include areas in which there may be existing laws and regulations that:

• are obesogenic (i.e. create an environment that contributes to obesity) e.g., land-use laws that allow for a large concentration of fast-food outlets selling energy-dense foods, and agricultural subsidies that result in an over-supply of sugar;

• serve as barriers to efforts to prevent obesity e.g., public liability laws as a barrier to opening school grounds after hours, and food safety laws that encourage packaged and not fresh food in pre-schools; and

• serve as facilitators to obesity prevention e.g., mandatory physical education in schools, car-free areas of cities.

The policy areas may also include areas in which there are regulatory gaps or weaknesses which, if addressed, would enhance obesity prevention efforts, for example, restricting food marketing to children, implementing front of pack nutrition signposting systems. Gaps and weaknesses may manifest themselves in the manner in which policy is implemented and interpreted in practice. The area of urban planning is a good illustrative example here. While in some cases urban planning legislation mandates that health be considered in planning decisions, this can potentially be overruled in practice by more specific legislation pertaining to other aspects of design and planning. Furthermore, the lack of enforceable guidelines on how to include health requirements in planning decisions may lead to the requirements being overlooked in some cases.

The structure of the tables highlights that multiple sectors and multiple levels of government have a role to play in efforts to prevent obesity, and may be useful in providing additional clarity as to the areas in which sectors beyond the health sector can play a role. While this article has only considered policy options of a regulatory nature, the same structure could be used to systematically identify opportunities for use of other policy instruments, such as programs and funding allocations. It is envisaged that the structure presented in this article can be easily adapted to apply to most countries around the world. As the tables are used in different policy contexts, the robustness of the structure will be tested, and there will be opportunity for the structure to be incrementally refined. Furthermore, the use of the two-dimensional analysis grids could be extended to identify obesity prevention policy opportunities beyond those targeting the food system and physical activity environments, such as those influencing other determinants of health and those supporting health services and clinical interventions. This may be valuable in considering a more holistic approach to obesity prevention.

## Conclusion

This article is intended to facilitate a systematic approach to identifying obesity prevention policies targeting the food system and physical activity environments. The analysis grids could provide a valuable tool for all levels and sectors of governments to use in developing and implementing their obesity prevention policy and actions. The broad array of potential regulatory policy interventions indicates the need to collect and evaluate evidence of the likely effectiveness of a range of interventions in order to inform an evidence-based prioritisation process.

The article highlights that a coordinated approach to policy development and implementation across all levels of government is necessary to deliver complementary policy action. Similarly, a collaborative 'whole of government' approach, spanning multiple-sectors, is required to avoid fragmented, overlapping or contradictory policies.

Where the structure presented in this article is used to map the policy environment and identify potential policy areas for intervention, this represents only an initial step in the overall process of bringing about policy change and subsequent implementation. The next steps as part of a systematic process are likely to be:

1. Working with relevant stakeholders to prioritise policy areas at each level of government to devise a prioritised short-list of potential intervention areas.

2. Analysing each of these potential intervention areas to understand the policy area in detail, including historical influences and constraints on policy change.

3. Defining specific interventions, and modelling their likely health and economic impacts, using the best available evidence to evaluate their effectiveness and cost-effectiveness.

## Competing interests

The authors declare that they have no competing interests.

## Authors' contributions

GS jointly developed the major concepts and drafted the manuscript. BAS jointly developed the major concepts and reviewed the manuscript. MAL jointly developed the major concepts and reviewed the manuscript. All authors read and approved the final manuscript.
